# Application of a targeted-enrichment methodology for full-genome sequencing of Dengue 1-4, Chikungunya and Zika viruses directly from patient samples

**DOI:** 10.1371/journal.pntd.0007184

**Published:** 2019-04-25

**Authors:** Uma Sangumathi Kamaraj, Jun Hao Tan, Ong Xin Mei, Louise Pan, Tanu Chawla, Anna Uehara, Lin-Fa Wang, Eng Eong Ooi, Duane J. Gubler, Hasitha Tissera, Lee Ching Ng, Annelies Wilder-Smith, Paola Florez de Sessions, Timothy Barkham, Danielle E. Anderson, October Michael Sessions

**Affiliations:** 1 Duke-NUS Medical School, Singapore; 2 National University of Singapore, Saw Swee Hock School of Public Health, Singapore; 3 Ministry of Health, Epidemiology Unit, Colombo, Sri Lanka; 4 Environmental Health Institute, Singapore; 5 Nanyang Technological University, Lee Kong Chian School of Medicine, Singapore; 6 Genome Institute of Singapore, Singapore; 7 Tan Tock Seng Hospital, Singapore; 8 National University of Singapore, Department of Pharmacy, Singapore; Oregon Health and Science University, UNITED STATES

## Abstract

The frequency of epidemics caused by Dengue viruses 1–4, Zika virus and Chikungunya viruses have been on an upward trend in recent years driven primarily by uncontrolled urbanization, mobility of human populations and geographical spread of their shared vectors, *Aedes aegypti* and *Aedes albopictus*. Infections by these viruses present with similar clinical manifestations making them challenging to diagnose; this is especially difficult in regions of the world hyperendemic for these viruses. In this study, we present a targeted-enrichment methodology to simultaneously sequence the complete viral genomes for each of these viruses directly from clinical samples. Additionally, we have also developed a customized computational tool (BaitMaker) to design these enrichment baits. This methodology is robust in its ability to capture diverse sequences and is amenable to large-scale epidemiological studies. We have applied this methodology to two large cohorts: a febrile study based in Colombo, Sri Lanka taken during the 2009–2015 dengue epidemic (n = 170) and another taken during the 2016 outbreak of Zika virus in Singapore (n = 162). Results from these studies indicate that we were able to cover an average of 97.04% ± 0.67% of the full viral genome from samples in these cohorts. We also show detection of one DENV3/ZIKV co-infected patient where we recovered full genomes for both viruses.

## Author summary

Dengue viruses 1–4 (DENV1-4), Zika virus (ZIKV) and Chikungunya virus (CHIKV) are tropical and subtropical viruses that share a common arthropod vector, and have very similar clinical presentations that are difficult to distinguish. With the recent outbreaks of DENV, ZIKV and CHIKV globally, a single methodology able to simultaneously distinguish these viruses and provide full-genome information would greatly increase our capacity to rapidly characterize outbreaks. As a proof of principle, we have applied this methodology to two large cohorts in Sri Lanka and Singapore taken during recent dengue and Zika outbreaks, respectively. Herein, we present the results of this application to these cohorts and provide the tools to replicate these methodologies for other cohorts.

## Introduction

Dengue viruses 1–4 (DENV1-4), Zika virus (ZIKV) and Chikungunya virus (CHIKV) are viruses spread by the *Aedes aegypti* and *Aedes albopictus* and are among the foremost arboviral threats to humans today [1]. DENV and ZIKV are flaviviruses with positive-sense, single-stranded RNA genomes of ~11 kb that encode for a single polyprotein, which is then post-translationally cleaved into three structural proteins (C, prM and E) and seven non-structural proteins (NS1, NS2A, NS2B, NS3, NS4A, NS4B and NS5) [2,3]. There are four different DENV serotypes (DENV1-4) that share 60–75% identity at the amino acid level. Collectively, DENV1-4 are responsible for an estimated 390 million infections yearly [4]. Evidence suggests that the geographic distribution of DENV is increasing [5] and poses a threat to travelers visiting affected regions [6].There is a single serotype of ZIKV that can be further broken down into two primary lineages, the African and the Asian/American. At the time of writing, ZIKV had spread to 86 countries [7], and is a documented cause of microcephaly in infants when transmitted vertically from an infected mother during pregnancy [8]. CHIKV is an alphavirus with three major lineages, all of which comprise a single serotype [9]. The genome of CHIKV is a positive-sense, single-stranded RNA genome of ~11.6 kb that is post-translationally cleaved into four nonstructural proteins (nsP1, nsP2, nsP3 and nsP4) and five structural proteins (C, E3, E2, 6k and E1) [9]. CHIKV has seen resurgence in the tropical and subtropical world with several notable epidemics in recent years [10].

Clinical presentation of infection with any of these six viruses is often similar; undifferentiated fever, headache, nausea/vomiting, persistent myalgia/arthralgia, and rash [11]. Although PCR assays that can discriminate between these viruses exist, they are often not used in conjunction and are reliant on short primer sequences designed to target relatively conserved regions of the viral genomes [12–15]. As these viruses all share a common replication strategy dependent on error-prone RNA-dependent RNA polymerases, rapid mutation necessitates the constant optimization of molecular diagnostic protocols for their accurate detection [12]. Further, these methodologies are limited in that they provide no information on the particular strain infecting the individual [12–15]. Recent studies have shown that polymorphisms in the viral genome can have profound effects on the pathogenicity and epidemic potential of the virus [16–22]. In response to these shortcomings, next-generation sequencing (NGS) is increasingly being used as a tool to obtain the full genome sequence of viruses in clinical samples [23]. However, the principle drawback to this methodology is the often-overwhelming amount of host material present in a clinical sample relative to the virus. In order to produce sufficient data for full viral genome assembly from these clinical samples, only a small number of samples can typically be run per lane of sequencing making this approach prohibitively expensive for many laboratories. To overcome the inherent limitations of this direct sequencing approach, a targeted enrichment approach to increase the sensitivity and efficiency of whole genome sequencing has been described for several pathogens of clinical importance [24–26]. One limitation of this approach is the high upfront cost associated with the enrichment baits. In the current study, we present a novel computation method, BaitMaker, to design baits that target the conserved and variable regions of a viral genome. The delineation of the conserved and variable regions is made possible by employing a computationally efficient k-mer based clustering approach on the available genetic information for DENV1-4, ZIKV and CHIKV in the NCBI database. We have then applied our methodology to two large cohorts: a collection of blood samples from the 2012–2015 DENV epidemic in Sri Lanka (n = 170) and samples collected during the 2016 outbreak of ZIKV in Singapore (n = 162) [27,28].

## Results

### BaitMaker: An algorithm to design minimal baits for targeted enrichment method

**Fig 1:**
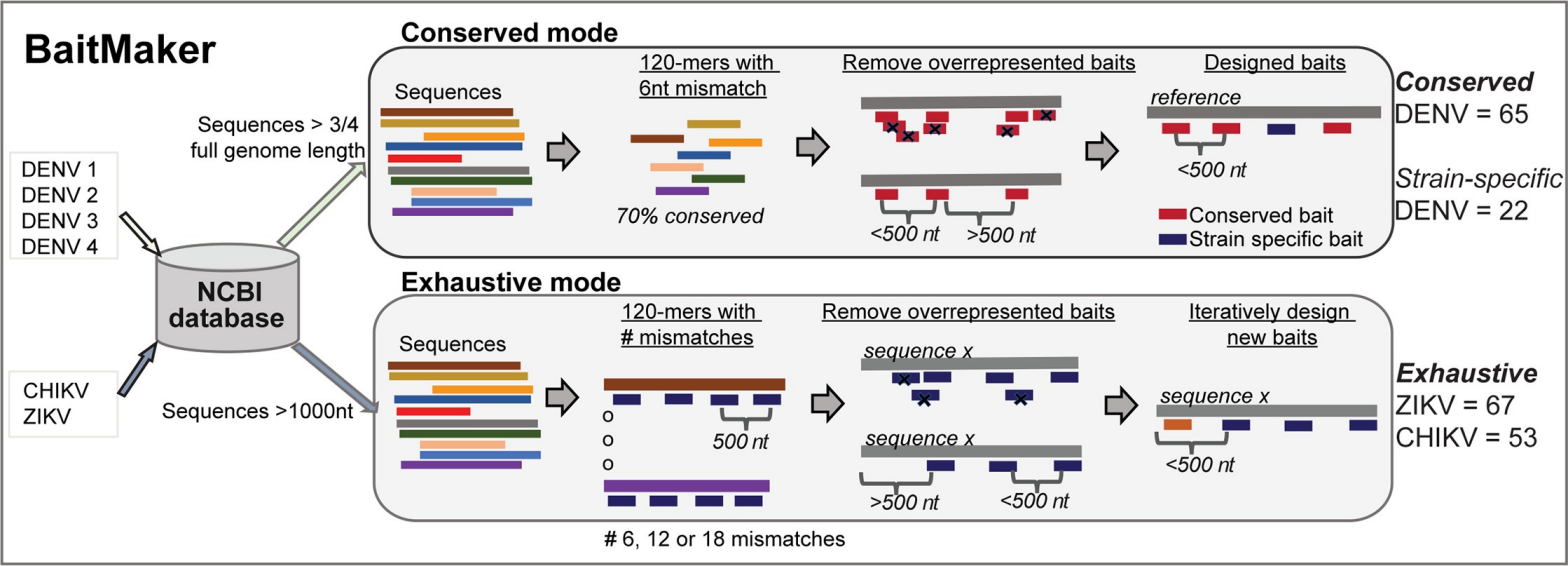
Overview of BaitMaker. BaitMaker has two modes, Conserved and Exhaustive mode to design 120 nucleotide (nt) baits for targeted genome enrichment methodology. (i) Conserved Mode: To generate conserved baits, complete or nearly complete viral genomes sequences were download from NCBI database. The mode generates baits for virus species at the region that is most conserved at species-level and present across the different strains. These conserved baits were identified by k-mer based search and clustering algorithm (PriMux) with the allowed number of six mismatches for bait hybridization. The baits were prioritized if the bait can target at least 70% of the sequences from NCBI. Assuming the sequencing library size is 300 nt, the overlapping baits within 500 nt were removed to get non-redundant conserved baits. For DENV, 65 conserved baits were generated. (ii) Exhaustive Mode: To generate exhaustive baits, viral sequences greater than 1000 nt were download from NCBI database. This mode generates all possible baits targeting all the sequences in the viral database and thus contains baits targeting all the different viral strains. Similar to the conserved mode, the overlapping baits and baits within 500 nt were removed. Then iteratively new baits were designed for the regions with no baits giving an exhaustive list of baits targeting all the genomic variation across different strains.

Targeted viral genome enrichment followed by high-throughput sequencing is an approach to enrich viral genomes present in meager quantities in clinical samples. The targeted-enrichment methodology uses biotinylated DNA baits, 120 nucleotides (nt) in length that are complementary to the viral genome. Commonly used algorithms to design baits generate tiled, overlapping baits across conserved genomic regions selected by multiple sequence alignment [24,29–32]. While effective, these methodologies can generate redundant and overlapping baits, which serves to increase the cost of the methodology in practice. In order to minimize the number of baits necessary to capture a target viral genome, we developed a new computational method called BaitMaker. BaitMaker generates non-overlapping baits at an interval of 500 nt in the viral genome. This interval was chosen under the assumption that an average deep sequencing library size ~300 nt and one bait can pull down two overlapping 300 nt DNA fragments. Hence, our approach differs from similar methodologies where placements of baits are tiled across the target genome [24,29–32] and allows for far fewer baits to be designed for each virus. In order to ameliorate the potential impact of reducing the number of baits available for capturing a targeted viral genome, BaitMaker incorporates a k-mer based pattern search and clustering strategy against a viral strain database (e.g. NCBI) to identify both conserved and diverse regions in the viral genome. BaitMaker then utilizes this information in one of two modes to design baits: (i) In ‘Conserved mode’, BaitMaker designs baits targeted to the species-level conserved regions whereas in (ii) ‘Exhaustive mode’, BaitMaker designs baits for both the conserved regions as well as regions with strain level variations. (**Fig 1 and Methods**). The source code for BaitMaker method is freely made available at GitHub: https://umasangumathi.github.io/BaitMaker/

For DENV serotypes 1, 2, 3 and 4, there were more than 11,000 genome sequences available in the NCBI database (**S1 Table**). Using BaitMaker-Conserved mode we generated conserved baits for each DENV serotype (65 baits total). As these baits are not designed to cover the genomic regions that are more variable, we designed an additional 22 baits that were specific to the Asian strains of DENV1-4 we were working with at the time (**S2 Table**). For ZIKV and CHIKV, there were fewer available sequences in the NCBI database at the time of design (238 and 1260, respectively) (**S1 Table**) hence, we developed the BaitMaker Exhaustive mode and utilized it to account for potentially under-represented variation in these viruses. The resulting panels for these viruses were 67 baits for ZIKV and 53 baits for CHIKV covering both conserved and variable regions of the respective genomes (**S2 Table).**

### Specificity and sensitivity of the targeted viral enrichment methodology

**Fig 2:**
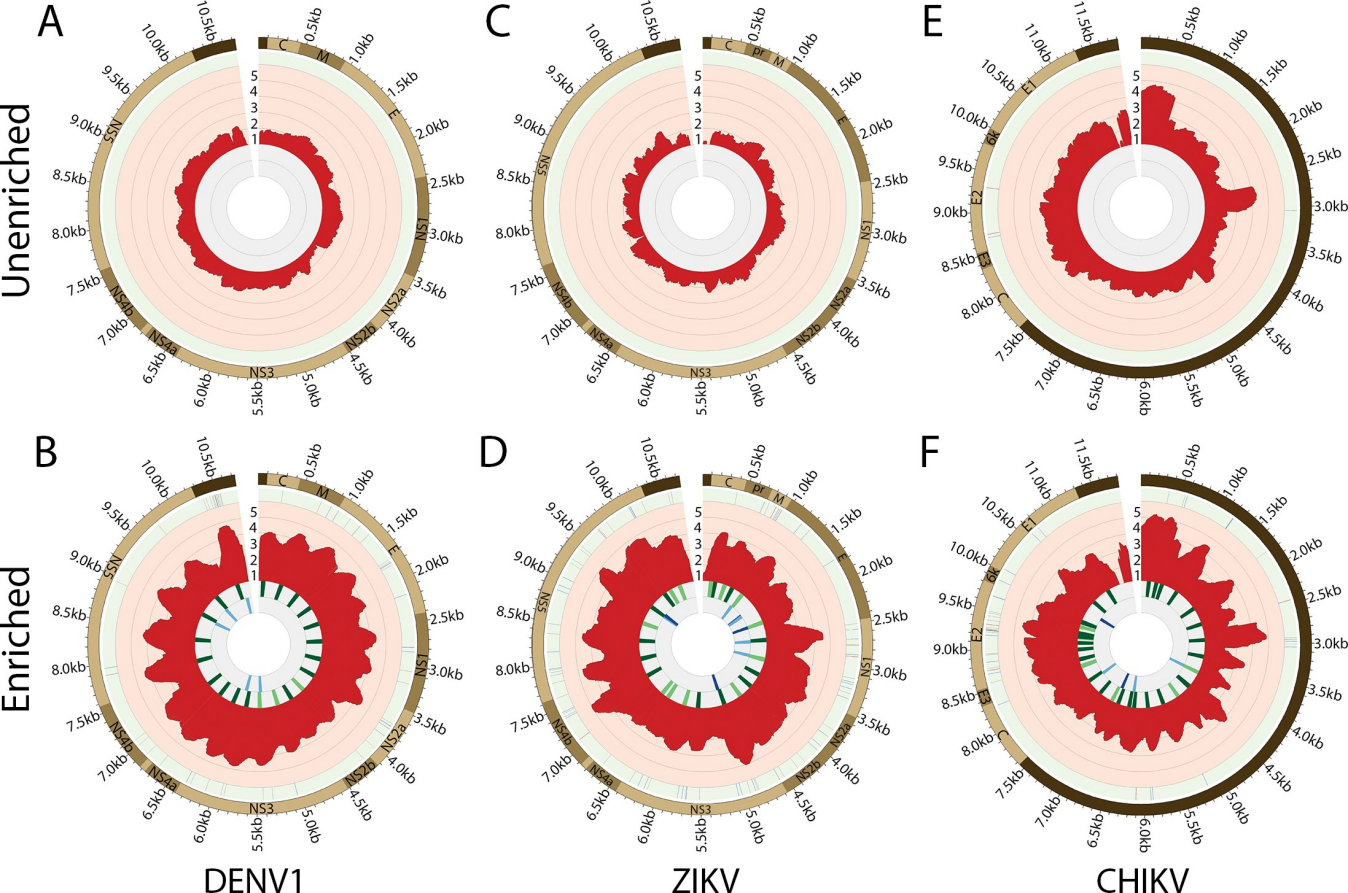
Genome coverage plots of unenriched and enriched samples of DENV1, ZIKV and CHIKV. The top panel (A, C, E) are unenriched samples whereas the bottom panel (B, D, F) are matched enriched samples with baits. From the outermost circle, each plot reads as the viral genes in the genome, SNPs (single nucleotide polymorphisms) detected, depth of coverage at each position in log scale shown in red and the baits hybridizing to the genome with varying sequence identity (80–85% identity in blue, 85–90% in dark blue, 90–95% in green and 95–100% in dark green). The number within the circle indicates the percentage of sequencing reads mapped to the genome.

To create a virus capture panel targeting DENV1-4, CHIKV and ZIKV simultaneously, we pooled the 87 baits (65 targeting the conserved regions, 22 targeting the variable regions) specific to DENV1-4, the 67 baits specific to ZIKV and the 53 baits specific to CHIKV for a total of 207 baits. To assess whether we could effectively capture the targeted viral genomes in the context of high host background, we infected HuH7 cells with DENV1, 2, 3 or 4, Vero cells with ZIKV and BHK-21 cells with CHIKV. To simulate a higher level of host contamination that would potentially be found in clinical samples, the supernatant was discarded from these cultures and total RNA was extracted from the infected cell monolayers and Illumina libraries were constructed from the total RNA. These libraries were then divided in half where the first half was sequenced directly, and the second half was enriched with our bait panel prior to sequencing. The resulting sequencing reads from both conditions were then mapped against the reference genome for each respective strain. Overall, we observed an average 2-log increase in percentage of sequencing reads mapped to the viral genome following enrichment in the DENV1-4, CHIKV and ZIKV samples (**Fig 2, S1 Fig and S3 Table**).

**Fig 3:**
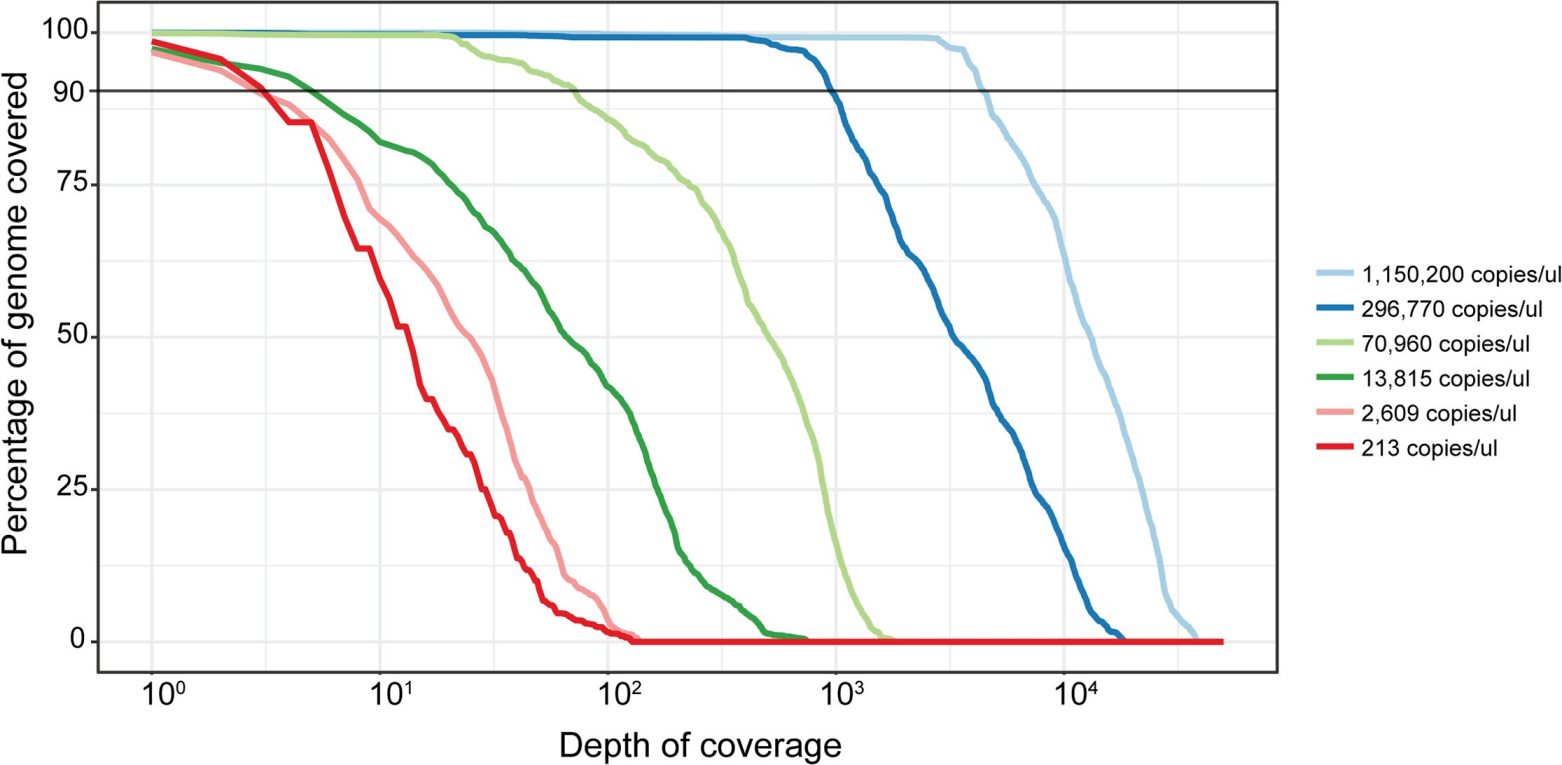
Effect of viral titers on the depth of coverage and breadth of genome covered in ZIKV dilution series. Ct indicates ZIKV viral titers of the samples. The x-axis represents the depth of coverage in log scale and y-axis represents the corresponding percentage of genome covered at the given coverage depth. The depth of coverage at 90% genome covered is discussed in the main text.

In order to assess the sensitivity of the assay, a 1:4 serial dilution of total RNA extracted from ZIKV infected Vero cells was prepared. In order to keep the level of host RNA constant, RNA extracted from uninfected Vero cells was used as the diluent. An approximate increase of two Ct values for each successive dilution with a range from 29.79 to 41.08 was observed for threshold detection. Libraries from these dilutions were then enriched and sequenced (**S3 Table)**. Our results indicate that as the Ct value increases, the depth of coverage decreases with respect to the breadth of genome coverage (**Fig 3).** In the sample with most virus (Ct value 29.79), 90% of the genome was covered with >4500x reads per genomic position. In the next dilution (31.57 Ct), this decreases to an average coverage of ~1000x reads per genomic position. Importantly, genome coverage at these depths is generally sufficient to assess inter/intra-host diversity in the virus population [33]. We also observe that the number of low frequency variants detected decreases as the dilution increases (**S3 Table**). At further dilutions (Ct values 33.45 and above), the average depth of coverage which covers 90% of the genome drops below ~100x. Although this level of coverage is generally considered to be insufficient for the estimation of viral subpopulations [33,34], consensus genomes can still be constructed and used for phylogenetic analyses.

### Application to clinical samples and the detection of viral co-infection

To measure the efficacy of the bait panel in clinical samples, we first tested the protocol on clinical samples representative of each of the different serotypes of DENV. These four samples were collected from DENV-infected patients who were enrolled in the early dengue infection and outcome (EDEN) study in Singapore [35,36] and from the DengueTools study in Sri Lanka [28]. The proportion of DENV reads relative to the host genome for different samples varied from 0.10% to 90.7%. After enrichment, the proportion of DENV-specific reads was between 94% and 99.6% for all serotypes tested. [**Table 1** and **S3 Table**]. The most successful enrichment occurred in the DENV4 sample, where only 0.10% DENV4 specific reads were present in the unenriched library. Following enrichment, the number of DENV4 specific reads was increased almost 3000-fold to 94% DENV4 specific sequences.

**Table 1:** Sequencing information of unenriched and enriched clinical samples with DENV infection.

Sample number	Clinical virus sample	Unenriched sample (% of reads mapped)	Enriched sample (% of reads mapped)	Log fold enrichment	Virus strain and NCBI sequence ID
1	05K4172 EDEN DENV1	77.45	99.6	0.11	DENV type 1 strain D1/SG/05K4172DK1/2005 (EU081261.1)
2	06K2352 EDEN DENV2	90.71	99.53	0.04	DENV2 strain BA05i (AY858035.2)
3	05K4176 EDEN DENV3	31.03	98.2	0.5	DENV type 3 strain D3/SG/05K4176DK1/2005 (EU081219.1)
4	SL558 Sri Lanka DENV4	0.10	94.18	2.98	DENV4 isolate DENV4/IND/0952326/2009 (JQ922560.1)

**Fig 4:**
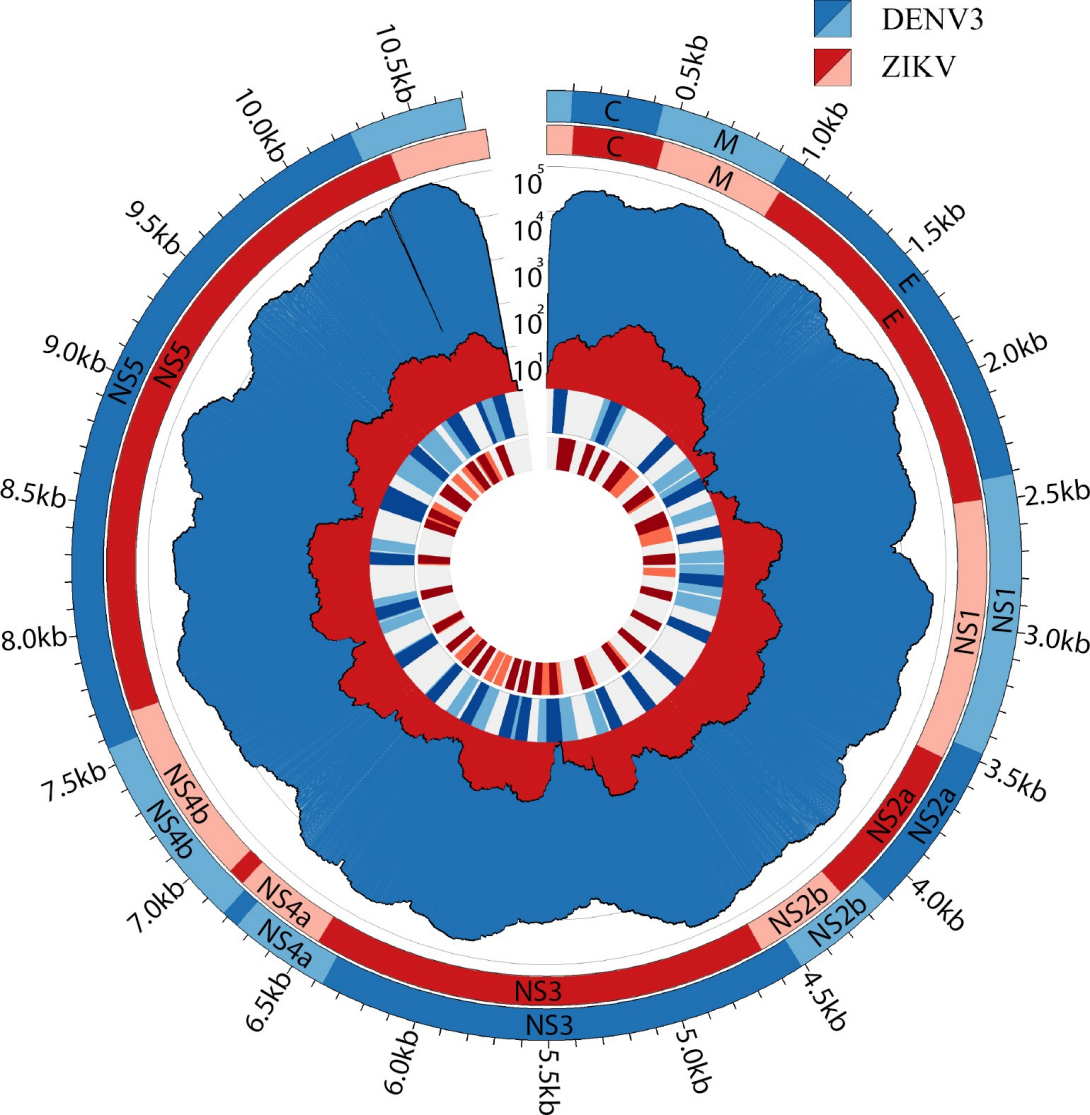
Co-infection of DENV3 and ZIKV identified in one clinical subject by using the DENV, CHIKV and ZIKV baits panel. From the outermost circle the plot reads as the DENV3 and ZIKV viral genomes, corresponding depth of coverage and the locations where the baits hybridize.

We then applied the bait panel to two clinical cohorts; one from Sri Lanka and another from Singapore. The first cohort was collected as a laboratory-based enhanced sentinel surveillance system in the Colombo District of Sri Lanka from 2012–2014 [28]. This collection period coincided with a severe dengue epidemic predominantly (~80%) driven by DENV1 with a smaller number (~20%) of cases due to DENV4 [28,37,38]. From this collection, we utilized our enrichment platform to obtain full or nearly full genome sequences for 143 DENV1 and 27 DENV4 isolates (**S2 Fig**). The second cohort of 162 samples was collected from August to September 2016 as part of a national response effort to the first outbreak of ZIKV in Singapore (**S2 Fig**) [27,39]. During the course of our investigation, we were also able to detect one dual infection in the Singaporean cohort where we were able to recover both DENV3 and ZIKV from the same patient (**Fig 4**).

To further examine the assumptions made by our method BaitMaker to generate baits, we investigated the baits’ features such as GC content, melting temperature, Gibbs free energy and sequence identity of the bait with the target genome and its effects on bait and target genome hybridization. We tested whether the baits’ features affected the baits’ pull-down efficiency, which is measured by the distribution of sequencing reads mapped to the genome around the bait. Based on the principal component analysis on the baits’ features and pull-down efficiency (**S3 Fig**) and effects of sequence identity on pull-down efficiency (**S4 Fig**), we observed that only sequence identity had an influence on pull-down efficiency and this efficiency decreases as the sequence identity between bait and target genome decreases. We next tested the effect of library size on the genome enrichment of the samples in both DENV and ZIKV cohorts (**S5 Fig**). We found that when the library size exceeds 300 nt, there is no further increase in coverage and this is expected as the baits were designed for library size of 300 nt. This further suggests that for larger library size, we can increase the bait design interval and thus only a smaller number of baits would be required to capture complete genomes. Thus, BaitMaker can efficiently design a set of minimal number of baits which is required to capture a complete genome.

## Discussion

DENV, ZIKV and CHIKV together represent the most significant arboviral threats to humans today with hundreds of millions of infections annually. These viruses are predominantly transmitted to humans by *Aedes aegypti* and *Aedes albopictus* species of mosquitoes whose global distribution has exploded in recent years, placing an estimated two-thirds of the world’s population at risk from contracting these diseases [4]. Calculating the true burden of disease for each of these viruses is complicated by not only overlapping clinical presentations but importantly by the fact that they co-circulate [11]. Given the commonality of the arthropod vectors these viruses employ for their transmission, the contribution of co-infection with these viruses to morbidity and mortality is poorly understood.

Full-genome sequencing of DENV1-4, ZIKV and CHIKV directly from clinical samples is not a routine practice in clinical, or even research, settings due to the costs associated with labor, reagents and time. Additionally, given the low success rate of current methodologies, loss of precious samples is a strong deterrent. Other groups have utilized a tiling approach wherein baits are designed to cover the entire length of a limited number of target genomes [24,29–32]. Although effective, we show here that this strategy represents an over-allocation of baits and is unnecessary to obtain full genome coverage of the target virus. Our BaitMaker program designs a minimal number of baits for viral capture that are non-overlapping and has the ability to capture the known variation in viral strains. Our approach significantly reduces the overall number of baits necessary to capture the diversity in these viruses which in turn minimizes the cost incurred and should assist in the broader implementation of this methodology.

Although we have currently only tested our methodology with Illumina sequencing, we foresee extending this methodology onto other sequencing platforms. Indeed, this methodology may have an even larger impact for the so called ‘third generation’ sequencing platforms (Pacbio and Nanopore) where average library sizes are far longer than Illumina but do not yield as many reads per run. Increasing the average library size would presumably translate to an even smaller number of baits required to capture full or near full genome coverage of the target virus as our results have shown strong positive correlation between library size and breadth of genome coverage (**S5 Fig**).

One particularly important benefit of utilizing a targeted enrichment approach over a Sanger sequencing or an unbiased metagenomic approach, is the number of reads specific to the viral genome are substantially increased and thus allows for assessment of intra-host genetic diversity. These data are particularly important for the analysis of low-frequency variants that may represent precursors to a change in overall pathogenicity [16,17]. As vaccines for these viruses are now in various stages of development, in-depth analysis of viral sub-populations will be critical to monitor vaccine escape mutants as they develop.

In this study, we have applied this methodology to a febrile cohort (n = 170) collected during a severe dengue outbreak in Sri Lanka and to a cohort (n = 162) collected during the 2016 Zika outbreak in Singapore. We were able to obtain full or nearly full genomes for the target viruses down to the limit of detection by qPCR. Importantly, we have combined bait panels for all four DENVs, ZIKV and CHIKV into a single assay and have detected a co-infection in a patient sample. Given the relatively small number of samples we have tested here and the overlapping clinical presentation of these viruses, the detection of a DENV/ZIKV co-infection indicates that the global prevalence of ZIKV and CHIKV could be higher than current estimates. Clinical management for DENV, ZIKV and CHIKV is largely supportive in nature with the vast majority of cases treated as outpatients and left to convalesce outside a clinical setting. Whether co-infection with these viruses is a predicator of adverse clinical outcome is largely understudied but it is a significant question that could potentially change clinical management and outcome for some of these patients [40]. In the one sample where we identified co-infection, the amount of DENV3 was much greater than ZIKV. Whether this result is due to differential viral kinetics, viral interference or temporal differences in the acquisition of each virus is an interesting question and is a subject of ongoing work.

Finally, we believe that application of the approach employed here for bait selection would potentially improve upon the large, pan-viral enrichment panels such as those recently published by Briese et al, 2017 and Wylie et al, 2015 [41,42]; with fewer baits required to achieve full genome coverage, more baits could be allocated to capture the known diversity in the targeted viral families. Increasing the amount of diversity in these panels would in turn increase the likelihood of capturing novel viruses in clinical and environmental samples.

## Materials and methods

### Ethics statement

Investigations described for the Zika samples were conducted as part of outbreak response operations by the Ministry of Health, Singapore to control the spread of Zika. Samples were taken opportunistically with verbal informed consent from subjects. Approval for the collection of dengue samples was obtained from the Ethics Review Committee, Faculty of Medicine, University of Colombo, Sri Lanka and informed consent obtained from participants in written format. Parental consent was obtained for study participants below 18 years of age. Data from both studies was anonymized prior to analysis and all methods were performed in accordance with the relevant guidelines and regulations.

### BaitMaker: Bait design algorithm

For targeted-enrichment method, we designed reverse complementary DNA baits of 120 nt in length, targeting the viral genome of interest. As the hybridization takes place at 65°C, we designed baits such that they had a melting temperature greater than this. The baits in our panel had melting temperature ranging from 69 to 87°C and GC content from 31 to 66%. We developed two modes to design baits (i) Conserved mode, to design baits at the species-level conserved regions and (ii) Exhaustive baits, to design baits for both conserved regions as well as regions with strain level variations.

#### Conserved mode

We downloaded available DENV1-4 sequences using the keyword search "txid12637 [Organism:exp] AND 1000:12000 [slen] NOT clone NOT cloning NOT vector NOT chimeric" and retrieved 11,152 sequences from NCBI. In order to retrieve full or nearly full genomes, we sub-selected the sequences with sequence length greater than 3/4^th^ of the maximum sequence length found for DENV in NCBI database. Some of the sequences deposited in NCBI are redundant and this may lead to overrepresentation of a specific sequence in our sub selected dataset. Hence, in order to get a diverse set of sequences representing DENV, we selected the sequences with at least two different values out of the seven metadata terms (sequence length, host, strain name, collection date, isolate name, country, strain taxonomic ID) to design baits. We then designed baits targeting the conserved regions using the PriMux software [43], a k-mer based search and clustering method to generate all possible overlapping 120 mers with a one nucleotide sliding window for the set of sequences within each DENV serotype. This k-mer approach ensures that for identical 120 mers present in different genome sequences, these 120 mers are grouped and a common 120 nt bait is designed to target them. As the hybridization process between the bait and viral DNA fragment is tolerant to mismatches, we permitted six mismatches out of 120 nt in the bait’s design. In NCBI, sequences less than full-genome length represent most of the known virus diversity, therefore, we prioritized the selection of the k-mers if it could target at least 70% of the NCBI sequences with at most six mismatches (95% identity). To further reduce redundancy in our bait design, we removed the 120 mers that overlapped or were within a 500 nt distance. This is based on the assumption that with an average library size of 300–500 nt, a 120 nt bait would be able to pull down at least two fragments of length 300 nt overhanging the 120 nt bait (300 nt + 300 nt -120 nt = 480 nt). Thereby, we designed 14 conserved baits for DENV1, 16 for DENV2, 19 for DENV3 and 16 for DENV4. However, few regions in DENV genome were highly variable, and therefore we designed 22 baits explicitly targeting a selected reference genome from South East Asian DENV cohort.

#### Exhaustive mode

To capture regions of greater diversity in the viral strains that cannot be captured by conserved baits, we developed “Exhaustive mode” to design baits targeting all the sequences in NCBI. We used the virus name and a minimum sequence length of 1000 nt to extract sequences from NCBI. In addition, we also removed sequence with these terms (clone, cloning, vector or chimeric) in the sequences’ metadata to ensure that the baits are not designed against vector sequence. For CHIKV and ZIKV, we designed baits such that they would capture all the known variations present in the viral strains, inclusive of partial genome sequences deposited in NCBI. The Exhaustive mode of BaitMaker is an iterative process; first, 120 nt baits complementary to genome spaced at a distance of 500 nt for each NCBI sequence are generated. After the initial baits are designed, CD-HIT [44], is employed to cluster and remove redundant baits. As hybridization remains efficient with even with a degree of mismatching between the bait and target sequences, we clustered the baits within a mismatch threshold of 18 nucleotides (85% identity) and 12 nucleotides (90% identity) for CHIKV and ZIKV, respectively. For remaining regions with no baits, the process was iteratively repeated until all regions within each target sequence was covered. A final pass of CD-HIT is then run to remove any redundancy in the bait panel.

In the last step of the algorithm, the baits are then checked for potential cross-hybridization with human, bacterial and mouse genomes using Blast. Any baits that are predicted to bind to host genomes were removed, and a new bait is selected for this region.

### Viral culture and clinical sample preparation

DENV1-4 and ZIKV were cultured in 2x10^5^ HuH7 and Vero cells, respectively, for 48 h or until cytopathic effects were observed. CHIKV was cultured in 2x10^5^ BHK21 cells for 20 h. After incubation, supernatant was removed and cell layers were scrapped into 250 μl of sterile PBS. RNA from serum and urine samples were extracted directly. All viral cultures and clinical samples were handled in a Class II-A2 biosafety cabinet under BSL-2 conditions according to national regulations pertaining to the handling of infectious agents.

### RNA extraction

RNA extraction was done using TRIzol Reagent (Life Technologies) according to the manufacturer’s instructions. Briefly, 250 μL of sample was added to 750 μL of TRIzol Reagent in a Phase Lock Gel Heavy 2 mL tube (5 PRIME) and incubated for 10 min at room temperature. Following incubation, 200 μL of chloroform (Sigma-Aldrich) was added and the mix was incubated for a subsequent 10 min. The sample was centrifuged for 5 min at 13,000 rpm. The aqueous phase was decanted into a new tube and 2 μL of Glycoblue Coprecipitant (ThermoFisher Scientific) and 500 μL of 2-propanol (Merck) was added to precipitate the RNA. The sample was centrifuged at 13,000 rpm for 15 min to obtain an RNA pellet. The pellet was washed with 500 μL of 75% ethanol (Merck), centrifuged for 3 min at 13,000 rpm, air-dried and resuspended in nuclease free H_2_O.

### Real-time PCR

Quantitative PCR (qPCR) was performed according to established methodologies [13]. Briefly, we used the QuantiTect Probe RT-PCR Kit (Qiagen) reagents and the CFX96 Real-Time System (Bio-Rad) where each 25 μL PCR reaction contained 12.5 μL 2X QuantiTect PCR mastermix, 1 μL of each 10 mM primer, 0.5 μL 0.2 mM probe, 0.5 μL reverse transcriptase, 3 μL RNA template and 6.5 μL H_2_O. Every PCR was performed as follows: reverse transcription at 50°C for 30 min, initial PCR activation at 95°C for 5 min and 45 amplification cycles consisting of a 95°C denaturation for 10 sec and a 60°C annealing/extension for 30 sec. Sequences of primers and probes are as follows; ZIKV-F: 5'- TGG TCA TGA TAC TGC TGA TTG C -3', ZIKV-R; 5'- CCT TCC ACA AAG TCC CTA TTG C -3', ZIKV-probe5'- /56-FAM/CGG CAT ACA GCA TCA GGT GCA TAG GAG /3BHQ_1/ -3', Vero (African green monkey) GAPDH-F:5′- GGG TGT GAA CCA TGA GAA GTA T-3′, GAPDH-R; 5′- GAG TCC TTC CAC GAT ACC AAA G-3′ and GAPDH-probe: 5'- /5HEX/AC AAC AGC CTC AAG ATC GTC AGC A/3BHQ_1/ -3'. The relative amount of viral transcript to GAPDH was calculated using the 2^-ΔΔ^CT method. Data were expressed as fold change RNA compared to the control.

### Preparation of Illumina DNA libraries from viral RNA

Illumina libraries were constructed from total RNA using NEBNext Ultra Directional RNA Library Prep Kit for Illumina (New England Biolabs) in conjunction with NEBNext Multiplex Oligos for Illumina (New England Biolabs) according to the manufacturer’s instructions with minor modifications. Briefly, 5 μL of total RNA was added to first strand synthesis buffer and random primers before incubating at 94°C for 2 min in order to generate RNA fragments larger than 500 nt. Following first strand and second strand cDNA synthesis, double-stranded cDNA was purified using Mag-Bind RxnPure Plus beads (Omega Bio-Tek) and eluted in 60 μL nuclease-free water. In order to obtain a library size between 400–600 nt, size selection of the libraries was performed using Mag-Bind RxnPure Plus beads (Omega Bio-Tek) in a two-step selection, by adding 35 μL, then subsequently 15 μL of beads to the reaction. The library was eluted in 20 μL nuclease-free water and amplified by PCR. Libraries were purified using the MinElute PCR Purification Kit (Qiagen), eluted in 25 μL nuclease-free H_2_O and visualized on a 1.5% agarose gel and quantified using a Bioanalyzer High Sensitivity DNA Assay (Agilent).

### Enrichment of viral library

Targeted viral enrichment was achieved using custom designed biotinylated, 120mer xGen Lockdown baits (Integrated DNA Technologies). Prior to capture of viral sequences, 1 μL each of xGen universal blocking oligo TS-p5 and TS-p7 (Integrated DNA Technologies), matched accordingly to the library index was added to 20 μL of library DNA and 0.5 μL of 5 μg Cot-1 DNA (Invitrogen) to block binding of baits to non-viral regions of library fragments. Blocked libraries were ethanol precipitated and resuspended in 2.5 μL H_2_O, 3 μL Nimblegen hybridization solution and 7.5 μL Niblegen 2X hybridization buffer (Roche). Following a 10 min incubation at room temperature, resuspended libraries were denatured at 95°C for 10 min and cooled on ice before the addition of the DENV, CHIKV and ZIKV bait pool. A total amount of 3 pmol of baits were added and hybridized to the libraries for 4 h at 65°C. To capture virus specific library fragments, 100 μL magnetic M-270 streptavidin Dynabeads (Life Technologies) were added to the hybridization reaction and the mix was incubated for a further 45 min at 65°C, with shaking at 2000 rpm in a ThermoMixer C (Eppendorf). Streptavidin beads were washed to remove unbound DNA using SeqCap EZ hybridization and wash kit (Roche) according to the manufacturer’s instructions. A post-capture PCR amplification of 20 cycles with P1 and P2 primers (Illumina) was performed and the enriched library was purified using the MinElute PCR Purification Kit (Qiagen). The purified, enriched library was eluted in 25 μL nuclease-free H_2_O, visualized on a 1.5% agarose gel and quantified using a Bioanalyzer High Sensitivity DNA Assay (Agilent). For the complete protocol, please see **S1 File**.

### Analysis of sequencing data

Enriched and unenriched libraries were constructed and sequenced on an Illumina MiSeq (Duke-NUS Genome Biology Facility, Singapore) and Illumina HiSeq 4000 (Genome Institute of Singapore). FastQC [45] was used to confirm the quality of the reads generated, and Trim Galore [46] was used to trim and filter the reads with a minimum quality cutoff of 20 and a minimum read length of 35 nt. As the viral species and strain is unknown in most of the cases, it is necessary to identify the nearest species and the strain present in the sample. Therefore, Blast toolkit [47] was used to search the nearest hit in the NCBI nucleotide database for every read using the megablast option. A metagenomic analysis software MEGAN [48] was used to cluster reads at the species level to visualize. As Blast analysis is time-consuming, only a portion of the reads were used to identify the species and strain. The species cluster with the maximum number of reads assigned was selected as the initial reference strain and used to generate a consensus genome. The consensus genome was generated by using bam2cons_iter.sh script from the ViPR pipeline [49]. The bam2cons_iter.sh uses BWA [50] to perform iterative mapping of the reads to the reference genome and a consensus is generated based on the maximum frequency of a nucleotide at a given position. From the obtained consensus genome, the nearest NCBI hit is found and used as a reference genome to rerun the bam2cons_iter.sh script with default parameters. This iterative consensus genome generation approach enables generation of a full genome consensus for the virus present in the sample. For final mapping with BWA mem v0.7.5 aligner was used to map the reads to the consensus reference genome and picard tools v1.95 [51] were used to mark PCR duplicates. Base calibration and indel realignment was done by GATK v3.3 [52]. Single nucleotide variants for each sample were detected using LoFreq2 software [33], which incorporates base-call quality scores as error probabilities into its model to distinguish SNVs from the average sequencing error rate, and assigns a p-value to each position (Bonferroni-corrected p-value > 0.05). LoFreq has previously been applied to DENV datasets, and its SNV predictions on these datasets have been experimentally validated down to 0.5% allele frequency [33], hence we filtered the SNPs with a threshold of coverage (>1000) and allele frequency (>0.5%). Finally, the genome coverage graph along with the baits positions and SNP positions were plotted using Circos [53].

The Pearson product-moment correlation analysis between the mean library size and one-standard deviation of Gaussian distribution was performed in R v3.3. The mean library size of the sample was computed using Picard-tools. The average one-standard deviation of Gaussian distribution per bait (>95% identity), was calculated by fitting a Gaussian distribution to the genome coverage in a window of 480nt around the bait. Quickfold from the mfold [54] package was used to find the Gibbs free energy for DNA bait secondary structure formation at temperature 65°C, 1 mM Na and 0 mM Mg. The principle component analysis between the GC content, melting temperature, identity and mean coverage at the region where the bait hybridizes with the genome was carried out in R.

## Supporting information

S1 Fig Genome coverage plots of unenriched and enriched samples of DENV2, DENV3 and DENV4. The top panel (A, C, E) are unenriched samples whereas the bottom panel (B, D, F) are matched enriched samples with baits. From the outermost circle, each plot reads as the viral genes in the genome, SNPs (single nucleotide polymorphisms) detected, depth of coverage at each position in log scale shown in red and the baits hybridizing to the genome with varying sequence identity (80–85% identity in blue, 85–90% in dark blue, 90–95% in green and 95–100% in dark green). The number within the circle indicates the percentage of sequencing reads mapped to the genome.
(TIF)

S2 Fig The mean genomic coverage of the clinical samples enriched by the DENV, CHIKV and ZIKV baits panel: A) 143 DENV1 samples, B) 27 DENV4 samples and C) 162 ZIKV samples. The standard error is represented as a lighter shade around the mean at each genomic location.
(TIF)

S3 Fig In order to ascertain if the other properties of the bait affected the affinity of the baits and fragments, we performed a principle component analysis (PCA) with the baits’ GC content, melting temperature (Tm), Gibbs free energy, sequence identity between bait and genome, and bait pull down efficiency measured by mean genome coverage. The samples from DENV1 (n = 143), DENV-4 (n = 27), and ZIKV (n = 162) cohort was used for this analysis. The first PCA component contains baits‘ GC and Tm, which are highly correlated (loading score of -0.659 and -0.663, respectively). The second PC component contains sequence identity and mean genome coverage that are highly correlated (loading score of 0.723 and 0.673, respectively). This suggests that the bait’s pull-down efficacy in term of mean genome coverage depends only on the bait’s sequence identity with the target genome.
(TIF)

S4 Fig Stacked depth of coverage at regions were the baits hybridize with the genome. The enriched samples used in Fig 2 (consisting of DENV1, 2, 3 and 4, CHIKV and ZIKV) was used to group the baits and genome targets based on their nucleotide identity: A) >95% identity (n = 111), B) 90–95% identity (n = 32), C) 85–90% identity (n = 10) and D) 80–85% identity (n = 25). The x-axis represents a 480 nt window of the genome with the bait at the center and the y-axis represents the stacked depth of coverage. Baits designed for DENV1-4 are represented as D1, D2, D3 and D4. The 95–100% identity group has a symmetric, Gaussian distribution when compared to the rest of the groups. In contrast, at 80–85% identity, the baits do not as effectively pull down their target region which is evident by the skew in distribution away from the center of the bait. This skew in the distribution of reads depth around the bait is likely due to the influence of a neighboring bait with a higher percent identity to its target region. For example, in the DENV2 sample at the genomic region between 8800 and 9100, the Bait-018-D2 bait hybridizes with 97.5% identity and zika_bait-43 (bait designed for ZIKV) binds with 81.2% identity and they overlap by 20 nt. Hence, for the 80–85% identity group, which includes the DENV2 genomic region enriched by zika_bait-43, has a skew towards the neighboring bait (Bait-018-D2) as it has higher binding identity. It should be noted however, that although the ZIKV bait binds at a lower efficiency, it is contributing to the coverage of DENV.
(TIF)

S5 Fig Effect of library size on genome enrichment by the baits panel. The x-axis is the mean library size of the samples and y-axis represents the genome coverage distribution given by 1-SD (standard deviation) of the sequencing reads distribution enriched around the bait. The range of library sizes for DENV1, DENV4, and ZIKV cohort samples are between 80–374 nt (median 203), 38–379 nt (median 175) and 101–800 nt (median 329) respectively. The number of samples in each cohort is indicated by n. For each virus group (DENV1, DENV4 and ZIKV), the Pearson’s product-moment correlation coefficient is calculated and denoted by r and confidence interval around the correlation coefficient is represented by CI. For DENV1 and DENV4 samples, there is a very strong positive Pearson correlation between coverage distribution and library size as their library sizes were below 300 nt. In contrast, there is a weak positive correlation in the Singapore ZIKV outbreak cohort samples due to the larger library size and number of baits targeting ZIKV is relatively higher (at >85% identity, DENV1 = 22, DENV4 = 20, ZIKV = 50). The black line represents the LOWESS (locally weighted scatterplot smoothing) fitted to all the cohort samples. When the library size exceeds 300 nt, there is no further increase in coverage. This is expected as the baits were designed for sample library of 300 nt. This further suggests that larger the library size, we can increase the bait design interval and thus only a smaller number of baits will be required to capture full viral genomes efficiently.
(TIF)

S1 Table Genomic sequences extracted from NCBI used to design baits. (XLSX)

S2 Table Properties of baits used in the experiments. (XLSX)

S3 Table Sequencing information in detail for unenriched and enriched experiment (Fig 1), the ZIKV dilution series experiment (Fig 2) and a DENV2 dilution series experiment (Figure not shown). (XLSX)

S1 File Enrichment protocol used in experiments. (DOCX)
