# Supplementary figures and images for: Application of a targeted-enrichment methodology for full-genome sequencing of Dengue 1-4, Chikungunya and Zika viruses directly from patient samples
Source: PLoS Negl Trop Dis. 2019 Apr 25;13(4):e0007184. doi: 10.1371/journal.pntd.0007184 (PMC6504110; doi:10.1371/journal.pntd.0007184)

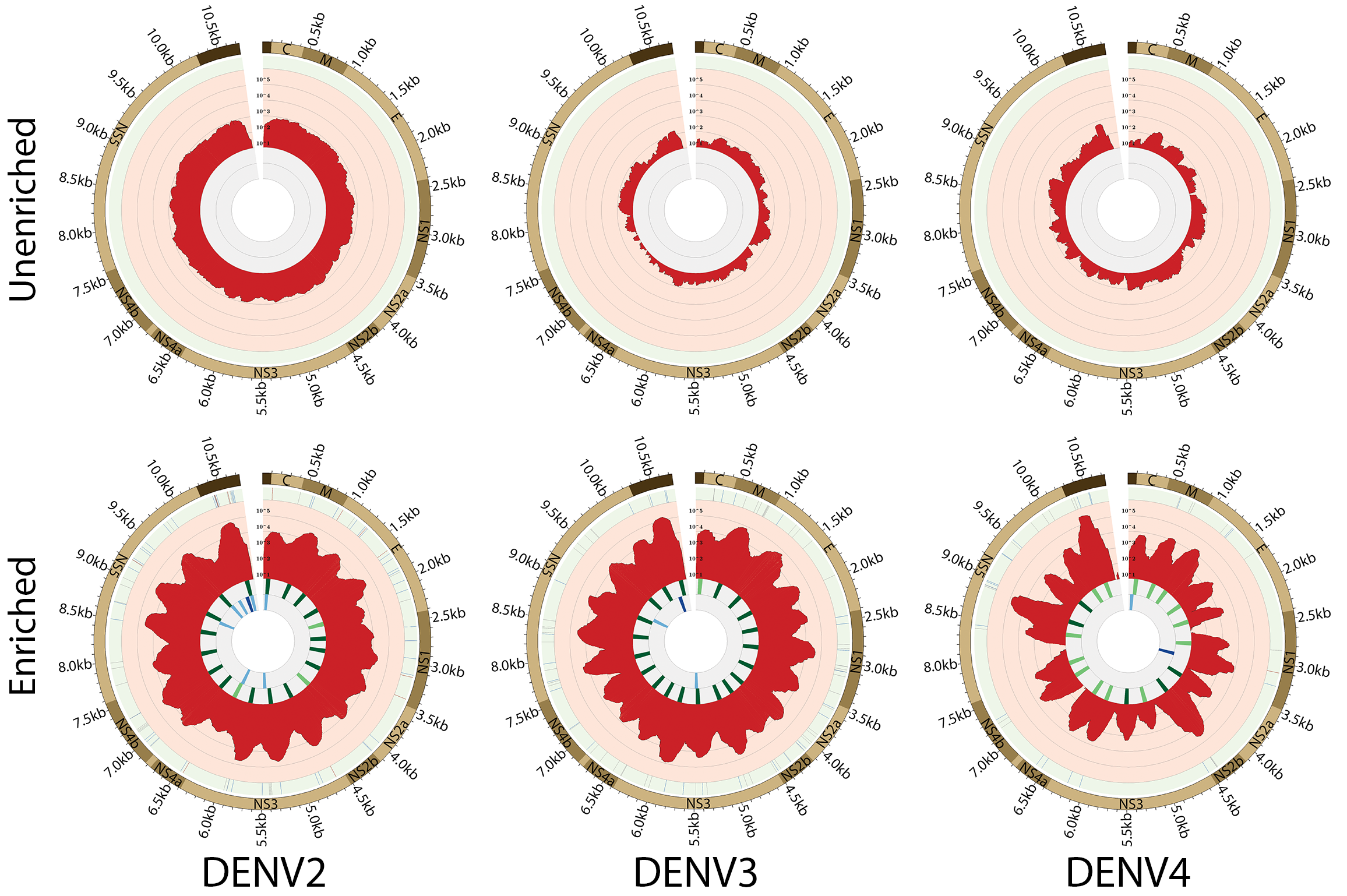

Supplement: S1 Fig — The top panel (A, C, E) are unenriched samples whereas the bottom panel (B, D, F) are matched enriched samples with baits. From the outermost circle, each plot reads as the viral genes in the genome, SNPs (single nucleotide polymorphisms) detected, depth of coverage at each position in log scale shown in red and the baits hybridizing to the genome with varying sequence identity (80–85% identity in blue, 85–90% in dark blue, 90–95% in green and 95–100% in dark green). The number within the circle indicates the percentage of sequencing reads mapped to the genome. (TIF) [file pntd.0007184.s001.tif]

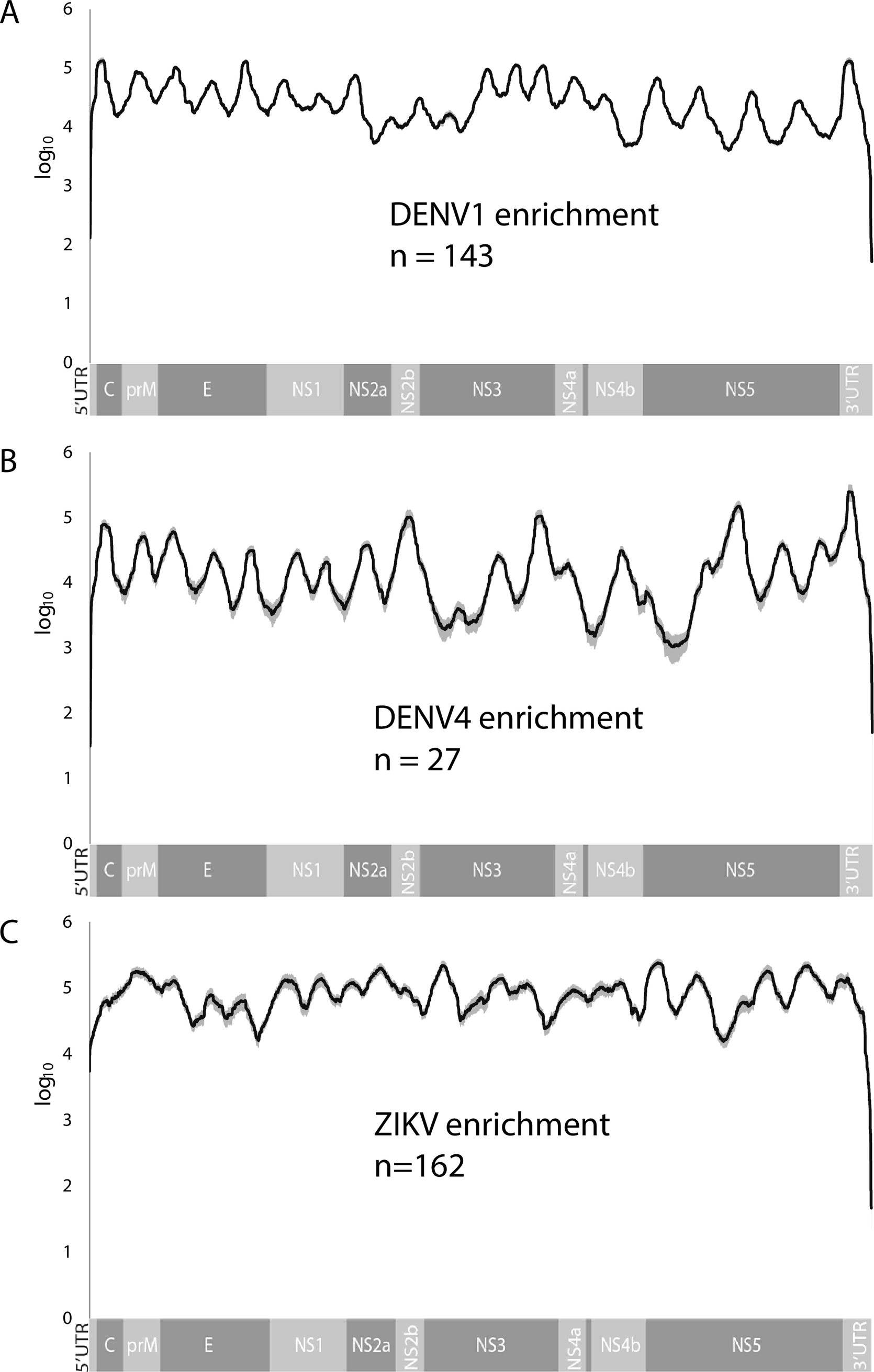

Supplement: S2 Fig — The mean genomic coverage of the clinical samples enriched by the DENV, CHIKV and ZIKV baits panel: A) 143 DENV1 samples, B) 27 DENV4 samples and C) 162 ZIKV samples. The standard error is represented as a lighter shade around the mean at each genomic location. (TIF) [file pntd.0007184.s002.tif]

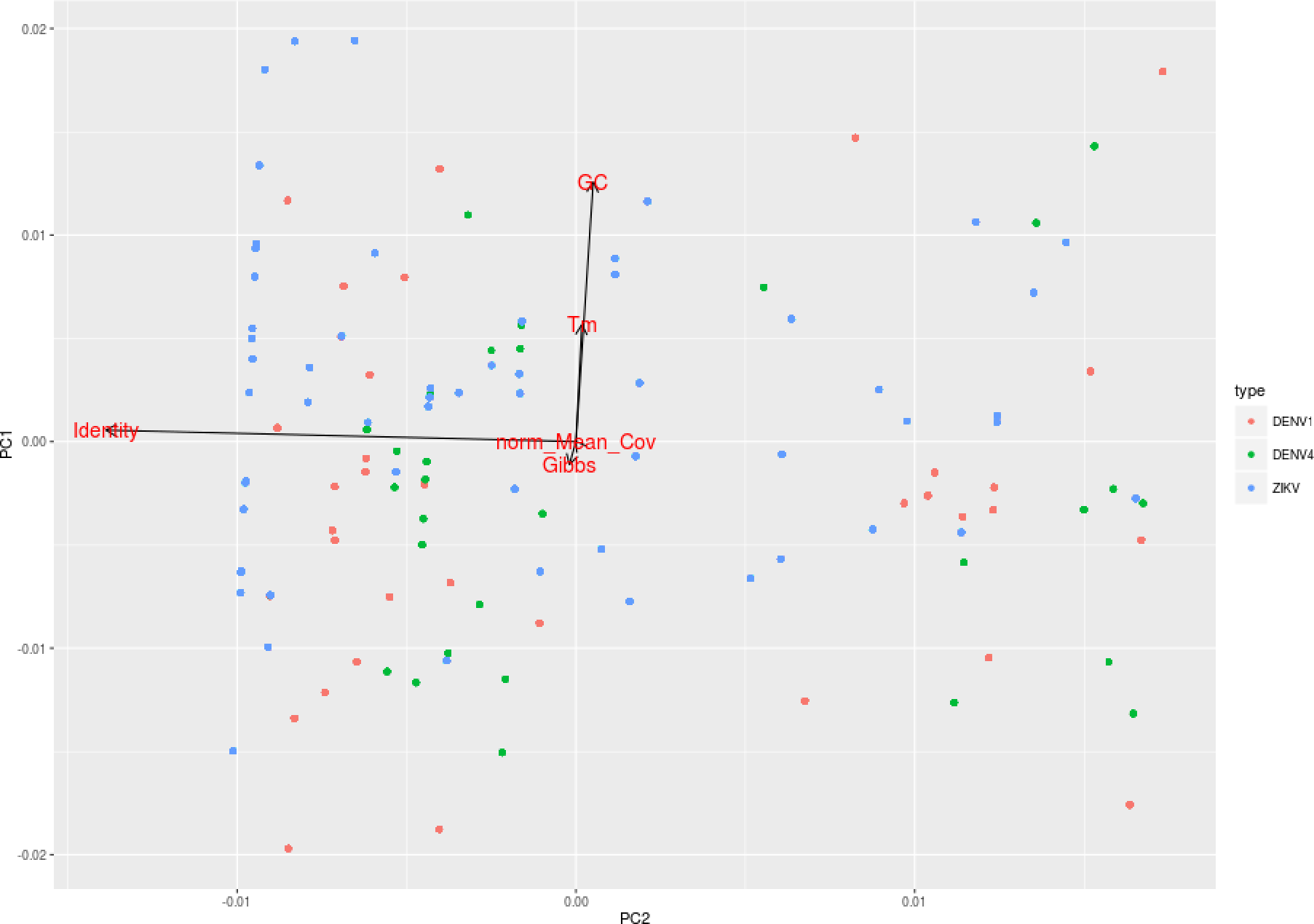

Supplement: S3 Fig — The samples from DENV1 (n = 143), DENV-4 (n = 27), and ZIKV (n = 162) cohort was used for this analysis. The first PCA component contains baits‘ GC and Tm, which are highly correlated (loading score of -0.659 and -0.663, respectively). The second PC component contains sequence identity and mean genome coverage that are highly correlated (loading score of 0.723 and 0.673, respectively). This suggests that the bait’s pull-down efficacy in term of mean genome coverage depends only on the bait’s sequence identity with the target genome. (TIF) [file pntd.0007184.s003.tif]

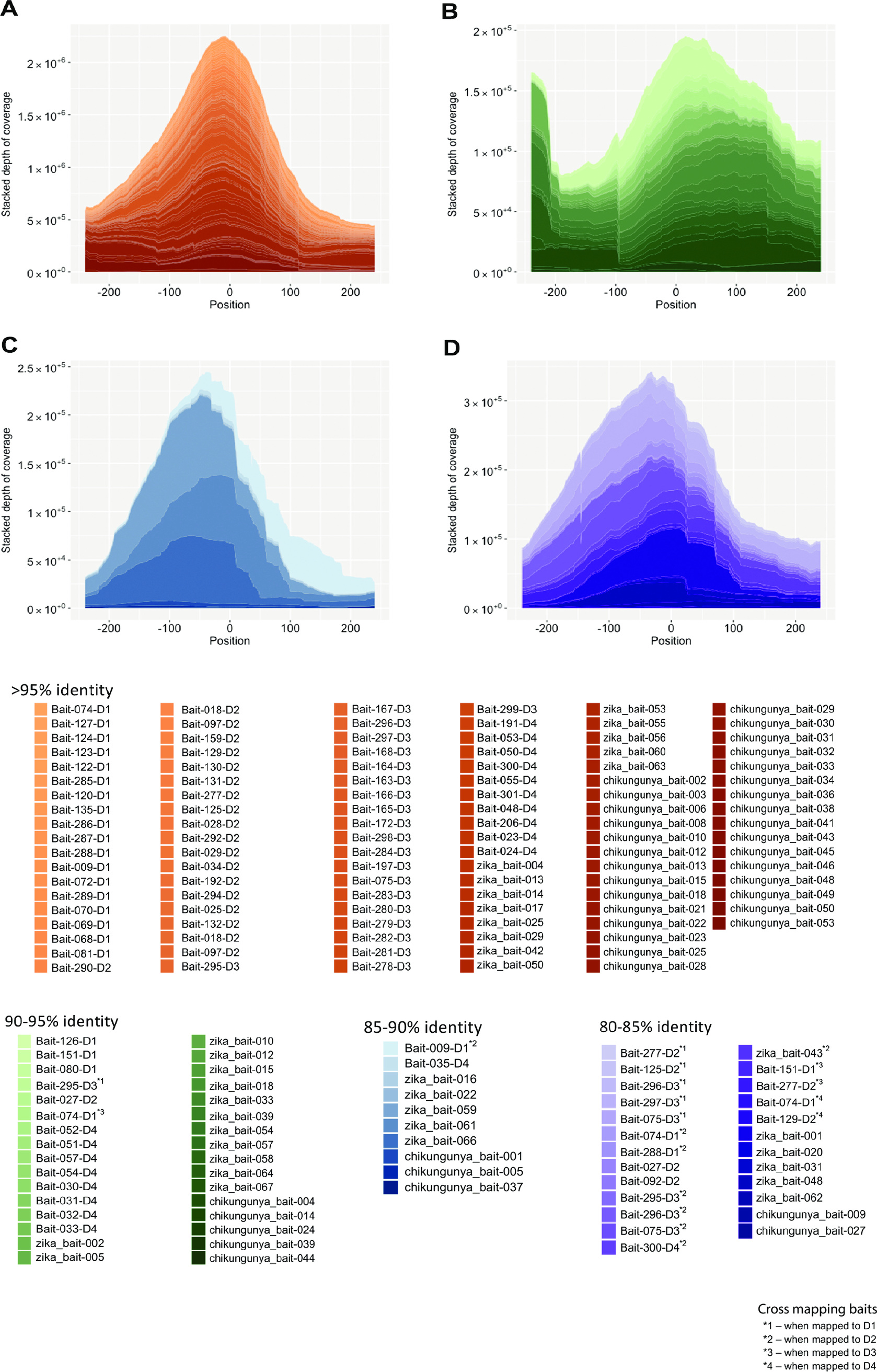

Supplement: S4 Fig — The enriched samples used in Fig 2 (consisting of DENV1, 2, 3 and 4, CHIKV and ZIKV) was used to group the baits and genome targets based on their nucleotide identity: A) >95% identity (n = 111), B) 90–95% identity (n = 32), C) 85–90% identity (n = 10) and D) 80–85% identity (n = 25). The x-axis represents a 480 nt window of the genome with the bait at the center and the y-axis represents the stacked depth of coverage. Baits designed for DENV1-4 are represented as D1, D2, D3 and D4. The 95–100% identity group has a symmetric, Gaussian distribution when compared to the rest of the groups. In contrast, at 80–85% identity, the baits do not as effectively pull down their target region which is evident by the skew in distribution away from the center of the bait. This skew in the distribution of reads depth around the bait is likely due to the influence of a neighboring bait with a higher percent identity to its target region. For example, in the DENV2 sample at the genomic region between 8800 and 9100, the Bait-018-D2 bait hybridizes with 97.5% identity and zika_bait-43 (bait designed for ZIKV) binds with 81.2% identity and they overlap by 20 nt. Hence, for the 80–85% identity group, which includes the DENV2 genomic region enriched by zika_bait-43, has a skew towards the neighboring bait (Bait-018-D2) as it has higher binding identity. It should be noted however, that although the ZIKV bait binds at a lower efficiency, it is contributing to the coverage of DENV. (TIF) [file pntd.0007184.s004.tif]

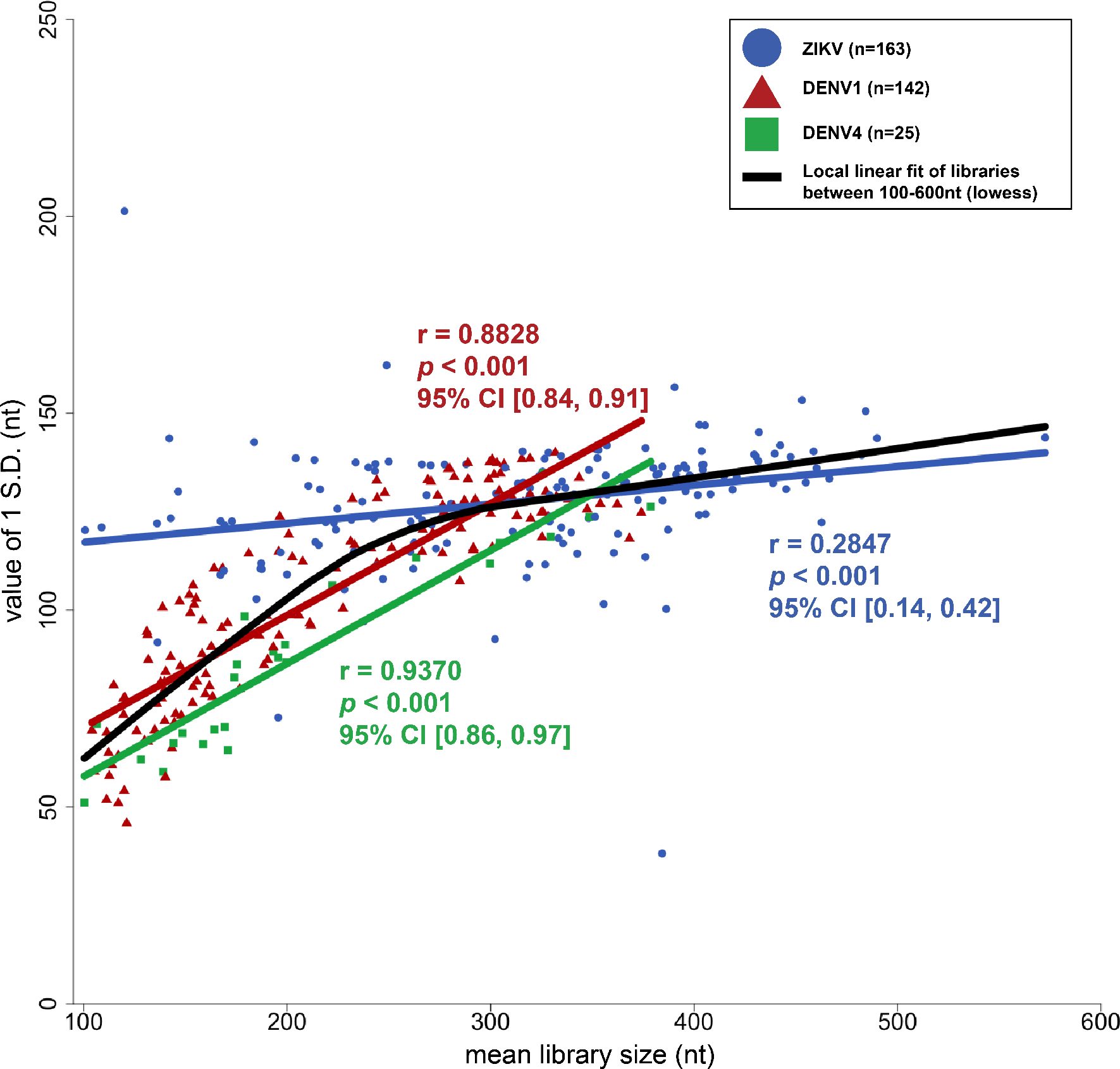

Supplement: S5 Fig — The x-axis is the mean library size of the samples and y-axis represents the genome coverage distribution given by 1-SD (standard deviation) of the sequencing reads distribution enriched around the bait. The range of library sizes for DENV1, DENV4, and ZIKV cohort samples are between 80–374 nt (median 203), 38–379 nt (median 175) and 101–800 nt (median 329) respectively. The number of samples in each cohort is indicated by n. For each virus group (DENV1, DENV4 and ZIKV), the Pearson’s product-moment correlation coefficient is calculated and denoted by r and confidence interval around the correlation coefficient is represented by CI. For DENV1 and DENV4 samples, there is a very strong positive Pearson correlation between coverage distribution and library size as their library sizes were below 300 nt. In contrast, there is a weak positive correlation in the Singapore ZIKV outbreak cohort samples due to the larger library size and number of baits targeting ZIKV is relatively higher (at >85% identity, DENV1 = 22, DENV4 = 20, ZIKV = 50). The black line represents the LOWESS (locally weighted scatterplot smoothing) fitted to all the cohort samples. When the library size exceeds 300 nt, there is no further increase in coverage. This is expected as the baits were designed for sample library of 300 nt. This further suggests that larger the library size, we can increase the bait design interval and thus only a smaller number of baits will be required to capture full viral genomes efficiently. (TIF) [file pntd.0007184.s005.tif]
