# Supplementary material for: Application of a targeted-enrichment methodology for full-genome sequencing of Dengue 1-4, Chikungunya and Zika viruses directly from patient samples
Source: PLoS Negl Trop Dis. 2019 Apr 25;13(4):e0007184. doi: 10.1371/journal.pntd.0007184 (PMC6504110; doi:10.1371/journal.pntd.0007184)
Supplement: S1 File — (DOCX) [file pntd.0007184.s009.docx]

**S1 File - Enrichment Protocol**

**Step 1 – Preparation of Lockdown Probes and Blocking Oligos (30 min)**

1. Hydrate the dried down pool of Lockdown® Probes to 1.5 pmol/μL in IDTE pH 8.0. If a capture probe pool at a lower concentration is used, IDT recommends drying down a portion of the material and rehydrating in water to 1.5 pmol/μL.

**Note- This step is only performed when the probes are first received.

1. Probes are at a concentration of 100 µM. Take 2 µl of each probe and add 18 µl IDTE to make a 1:10 stock of 10 µM.

Combine 1 µl of each 10 µM probe stock in a new Eppendorf. This is the stock probe mix.

Aliquote the probe stock mix into 20ul aliquots and store at -20°C.

3. For xGen® Universal Blocking Oligos, spin down and resuspend in 25 µl IDTE pH 8.0 for 25 reactions.

**Step 2 - Hybridization of DNA Lockdown Probes to the DNA Library (6.5 hr)**

The step is carried out according to xGen® Lockdown® Probes/Panels protocol.

**For Illumina TruSeq® LT Libraries (or other manufacturers using Illumina barcodes)**


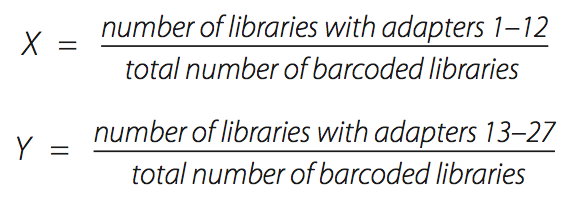
* Important: If you are using a combination of 6 nt (adapters 1–12) and 8 nt (adapters 13–27) barcoded TruSeq® LT adapters, use the formulas below to determine the fractions of 6 nt (X) and 8 nt (Y) blocking oligos you will need. X + Y must equal 1.

1. Combine the following in a low-bind 1.7 mL PCR tube:

| **Components** | **Volume (μl)** | **X11** |
| --- | --- | --- |
| 500 ng pooled, barcoded Illumina TruSeq® LT Libraries | 20 |  |
| 5 μg Cot-1 DNA (1mg/ml) | 0.5 | 5.5 |
| xGen® Universal Blocking Oligo – TS-p5 | 1 | 11 |
| xGen® Universal Blocking Oligo – TS-p7 (6 nt) | 1 | 11 |
| xGen® Universal Blocking Oligo – TS-p7 (8 nt) | 0 | 0 |
| **Total volume** | **22.5** | **2.5ea** |

2. Add the following components to the tube containing the library and blocking oligo:

| **Components** | **Volume (μl)** | **X12** |
| --- | --- | --- |
| Library and blocking oligos | 22.5 |  |
| 3M Sodium Acetate, pH 5.2 | 2 | 24 |
| Linear Acrylamide, 5 mg/ml | 4 | 48 |
| Glyco blue | 1 | 12 |
| 100% Ethanol | 60 |  |
| **Total volume** | **89.5** | **7ea** |

3. Incubate at -80°C for 30 minutes (or O/N).

4. Dilute 1 µl of stock probe mix in 5.7 µl IDTE buffer for a 1.5 pmol/µl solution. For more than 3 captures, refer to the table below for the dilution of the probe.

| Captures | Amount 10 µM probe mix (µl) | Amount IDTE buffer (µl) |
| --- | --- | --- |
| 1-3 | 1 | 5.7 |
| 4-6 | 2 | 11.4 |
| 7-9 | 3 | 17.1 |
| 10-12 | 4 | 22.8 |

5. Centrifuge at 14,000 rpm for 25 min at 4°C.

6. Carefully remove ethanol.

7. Wash pellet with 300 μl of 70% ethanol.

8. Centrifuge at 14,000 rpm for 5 min at 4°C.

9. Carefully remove 70% ethanol and air dry pellet for up to 10 min at room temperature.

10. Resuspend pellet in the following (leave the solution in the Eppendorf tube for 10 minutes to go into solution):

| **Components** | **Volume (μl)** | **X11** |
| --- | --- | --- |
| Nimblegen 2X Hybridization buffer (vial 5) | 7.5 | 82.5 |
| Nimblegen Hybridization Component A (vial 6) | 3 | 33 |
| Nuclease-free water | 2.5 | 27.5 |
| **Total volume** | **13** | **13ea** |

11. Transfer 13 µl reaction to a PCR tube and incubate in a thermal cycler at 95°C for 10 min.

12. Cool on ice and add 2 μl Lockdown Probe pool (3 pmol total) to the tube. Pipette to mix.

13. Incubate hybridization reaction at 65°C (set heated lid at 75°C) for 4 hours.

**Step 3 - Prepare Bead Wash Buffers (15 min)**

*Important: *The temperature of the heat block* ***must*** *remain at 65°C.*

1. Dilute 10X Wash Buffers (I, II, III, and Stringent) and 2.5X Bead Wash Buffer to create 1X working solutions.

| **Concentrated Buffer** | **Final volume 1X Buffer** | **Volume required (µl)** | **Volume H_2_O (µl)** | **Final volume 1X Buffer** | **Volume required (µl)** | **Volume H_2_O (µl)** |
| --- | --- | --- | --- | --- | --- | --- |
|  |  |  |  | **X12** | **X12** | **X12** |
| 10X Wash Buffer I | 300 | 30 | 270 | 3600 (1200*3) | 360 (120*3) | 3240 (1080*3) |
| 10X Wash Buffer II | 200 | 20 | 180 | 2400 (1200*2) | 240 (120*2) | 2160 (1080*2) |
| 10X Wash Buffer III | 200 | 20 | 180 | 2400 (1200*2) | 240 (120*2) | 2160 (1080*2) |
| 10X Stringent Wash Buffer | 400 | 40 | 360 | 4800 (1600*3) | 480 (160*3) | 4320 (1440*3) |
| 2.5X Bead Wash Buffer | 500 | 200 | 300 | 6000 (2000*3) | 2400 (800*3) | 3600 (1200*3) |

2. For each capture reaction, preheat the following wash buffers to 65°C in a heat block:

| **Components** | **Volume (μl)** | **X12** |
| --- | --- | --- |
| 1X Stringent Wash Buffer | 400 | 4800 (1600*3) |
| 1X Wash Buffer I | 100 | 1200 |

*Equilibrate buffers at 65°C for at least 2 hr before starting wash steps of the captured DNA.

**Step 4- Prepare Streptavidin Dynabeads (40 min)**

*Only begin this step when the hybridization reaction (Step 2) has 40 min remaining.

1. Allow Dynabeads M-270 Streptavidin to equilibrate to room temperature for 30 min before use.

2. Mix the beads thoroughly by vortexing for 15 sec.

3. Aliquot 100 μL streptavidin beads per capture into a single 1.7 mL low-bind tube (i.e., for 1 capture use 100 μL beads, for 2 captures use 200 μL beads. Up to 8 captures per tube).

| **Components** | **Volume (μl)** | **X12** |
| --- | --- | --- |
| Streptavidin beads | 100 | 600 (*2) |

4. Place the tube in a magnetic separation rack. Carefully remove and discard the supernatant ensuring that all of the beads remain in the tube.

5. Add 200 μL 1X Bead Wash Buffer per 100 μL beads. Vortex for 10 sec.

| **Components** | **Volume (μl)** | **X12** |
| --- | --- | --- |
| 1X Bead Wash Buffer | 200 | 1200 (*2) |

6. Place the tube in a magnetic separation rack. Carefully remove and discard the supernatant ensuring that all of the beads remain in the tube.

7. Add 200 μL 1X Bead Wash Buffer per 100 μL beads. Vortex for 10 sec.

| **Components** | **Volume (μl)** | **X12** |
| --- | --- | --- |
| 1X Bead Wash Buffer | 200 | 1200 (*2) |

8. Place the tube in a magnetic separation rack. Carefully remove and discard the supernatant ensuring that all of the beads remain in the tube.

9. After removing the buffer following the second wash, add 1X the original volume of beads of 1X Bead Wash Buffer (i.e., for 100 μL beads, use 100 μL buffer) and resuspend by vortexing.

| **Components** | **Volume (μl)** | **X12** |
| --- | --- | --- |
| 1X Bead Wash Buffer | 100 | 600 (*2) |

10. Transfer 100 μL of the resuspended beads into a new low-bind eppendorf tube for each capture reaction.

11. Place the tube in a magnetic rack to bind the beads. Allow the beads to separate from the supernatant. Carefully remove and discard the clear supernatant ensuring that all of the beads remain in the tube.

*Important: Proceed immediately to the next step. Do not allow the Dynabeads to dry out. Small amounts of residual Bead Wash Buffer will not interfere with downstream binding of the DNA to Dynabeads.

**Step 5- Bind hybridized target to the streptavidin beads (50 min)**

1. Transfer the 15 µl hybridization samples from Step 2 to the tube containing prepared streptavidin beads.

2. Mix thoroughly by pipetting up and down 10 times. Transfer the beads and buffer back into the same PCR tube.

3. Place the tube into a ThermoMixer (2000rpm) set to 65°C for 45 min to bind the DNA to the beads.

4. Quick spin the tubes every 15 min to ensure that the beads remain in suspension.

**Step 6- Wash streptavidin beads to remove unbound DNA (30 min)**

* Important: Work quickly to ensure temperature does not drop much below 65°C.

1. Add 100 μl pre-heated 1X Wash Buffer I to the tube and mix by pipetting.

2. Transfer the mixture to a fresh low-bind 1.7 mL tube.

3. Place the tube in the magnetic separation rack. Allow the beads to separate from the supernatant. Using a pipette, remove the supernatant containing unbound DNA and discard.

4. Add 200 μl preheated 1X Stringent Wash Buffer and pipette up and down 10 times to mix. Incubate at 65°C for 5 min.

5. Place the tube in the magnetic separation rack. Allow the beads to separate from the supernatant. Using a pipette, remove the supernatant containing unbound DNA and discard.

6. Add 200 μl preheated 1X Stringent Wash Buffer and pipette up and down 10 times to mix. Incubate at 65°C for 5 min.

7. Place the tube in the magnetic separation rack. Allow the beads to separate from the supernatant. Using a pipette, remove the supernatant containing unbound DNA and discard.

8. Add 200 μl room temperature 1X Wash Buffer I and vortex for 2 min to mix.

9. Place the tube in the magnetic separation rack. Allow the beads to separate from the supernatant. Using a pipette, remove the supernatant and discard.

10. Add 200 μl room temperature 1X Wash Buffer II and vortex for 1 min to mix.

11. Place the tube in the magnetic separation rack. Allow the beads to separate from the supernatant. Using a pipette, remove the supernatant and discard.

12. Add 200 μl room temperature 1X Wash Buffer III and vortex for 30 sec to mix.

13. Place the tube in the magnetic separation rack. Allow the beads to separate from the supernatant. Using a pipette, remove the supernatant and discard.

14. Remove the tube from the magnetic rack and add 20 μl Nuclease-Free Water to resuspend the beads. Mix thoroughly by pipetting up and down 10 times.

15. Transfer the 20 μl reaction (with the beads) to a new PCR tube.

**Step 7- Post-Capture PCR (1 hr 15 min)**

**1) Library Amplification Reaction Setup (45 min)**

1. Prepare the reaction mix.

| **Components** | **Volume (µl)** | **X12** |
| --- | --- | --- |
| 10X PFU UltraII buffer | 5 | 60 |
| 10 mM dNTPs | 0.5 | 6 |
| PCR Primer (10 μM Illumina P1 F primer) | 1 | 12 |
| PCR Primer (10 μM Illumina P2 R primer) | 1 | 12 |
| PFU UltraII DNA Polymerase | 1 | 12 |
| Enriched library DNA on beads | 20 |  |
| H_2_O | 21.5 | 258 |
| **Total volume per well/tube** | **50** | **30ea** |

2. Briefly vortex the mixture and quick spin. Place reactions in the thermocycler and run the following program:

| Step | Temp | Duration | Cycles |
| --- | --- | --- | --- |
| Initial denaturation | 95 °C | 2 min | 1 |
| Denaturation | 95 °C | 20 sec | 20 |
| Annealing | 65 °C | 20 sec |  |
| Extension | 72 °C | 15 sec |  |
| Final extension | 72 °C | 3 min | 1 |
| Stop reaction | 4 °C | Hold | Hold |

**2) Library Amplification Cleanup (30 mins)**

1.  Purify the library with beads using Qiagen MinElute kit.

2.  Elute DNA with 15μl water. This is the enriched NGS library.

3.  Run 2ul of the library on the LabChip for verification. **Note- if elution is done in TE buffer, this must be used to dilute the ladder for the LabChip.

4. Proceed with library QC or sequencing, as appropriate.

5. If necessary, re-amplify by repeating Step 7 (post-capture PCR and cleanup).
